# Effect of Sodium Bicarbonate Administration on Mortality in Patients with Lactic Acidosis: A Retrospective Analysis

**DOI:** 10.1371/journal.pone.0065283

**Published:** 2013-06-05

**Authors:** Hyun Jeong Kim, Young Ki Son, Won Suk An

**Affiliations:** 1 Department of Internal Medicine, Dong-A University College of Medicine, Busan, Korea; 2 Institute of Medical Science, Dong-A University College of Medicine, Busan, Korea; D'or Institute of Research and Education, Brazil

## Abstract

**Background:**

Lactic acidosis is a common cause of high anion gap metabolic acidosis. Sodium bicarbonate may be considered for an arterial pH <7.15 but paradoxically depresses cardiac performance and exacerbates acidosis by enhancing lactate production. This study aimed to evaluate the cause and mortality rate of lactic acidosis and to investigate the effect of factors, including sodium bicarbonate use, on death.

**Methods:**

We conducted a single center analysis from May 2011 through April 2012. We retrospectively analyzed 103 patients with lactic acidosis among 207 patients with metabolic acidosis. We used SOFA and APACHE II as severity scores to estimate illness severity. Multivariate logistic regression analysis and Cox regression analysis models were used to identify factors that affect mortality.

**Results:**

Of the 103 patients with a mean age of 66.1±11.4 years, eighty-three patients (80.6%) died from sepsis (61.4%), hepatic failure, cardiogenic shock and other causes. The percentage of sodium bicarbonate administration (p = 0.006), catecholamine use, ventilator care and male gender were higher in the non-survival group than the survival group. The non-survival group had significantly higher initial and follow-up lactic acid levels, lower initial albumin, higher SOFA scores and APACHE II scores than the survival group. The mortality rate was significantly higher in patients who received sodium bicarbonate. Sodium bicarbonate administration (p = 0.016) was associated with higher mortality. Independent factors that affected mortality were SOFA score (Exp (B) = 1.72, 95% CI = 1.12–2.63, p = 0.013) and sodium bicarbonate administration (Exp (B) = 6.27, 95% CI = 1.10–35.78, p = 0.039).

**Conclusions:**

Lactic acidosis, which has a high mortality rate, should be evaluated in patients with metabolic acidosis. In addition, sodium bicarbonate should be prescribed with caution in the case of lactic acidosis because sodium bicarbonate administration may affect mortality.

## Introduction

Lactic acidosis is defined as hyperlactatemia and metabolic acidosis with increased anion gap. Lactic acidosis, which is caused by the accumulation of lactate, is a common cause of high anion gap metabolic acidosis in severely ill patients in the hospital [1]. The common causes of lactic acidosis are associated with impaired tissue oxygenation due to shock. Therefore, severe septic, cardiogenic and hypovolemic shock may frequently result in lactic acidosis. In contrast, vasoconstrictors for increasing blood pressure may worsen tissue perfusion and aggravate lactic acidosis. Consequently, managing lactic acidosis is very difficult, and patients with lactic acidosis have a high mortality rate of 59 to 99% [2], [3]. Lactic acidosis is also commonly associated without oxygen deficits, such as toxins, drugs that include metformin, malignancy, hepatic failure, renal failure and diabetes mellitus. Correcting the underlying causes and managing the predisposing disorders are the most important interventions for lactic acidosis because there is no definite therapy for lactic acidosis [4].

Metabolic acidosis with lactic acidosis or without lactic acidosis may depress cardiac contractility and predispose patients to pulmonary edema by decreasing pulmonary vascular compliance. Correction of acidosis with sodium bicarbonate may reverse the depressed cardiac performance in critically ill patients. Therefore, sodium bicarbonate administration may be considered for an arterial pH <7.15 in patients with lactic acidosis because myocardial depression and diminished myocardial response to catecholamine at pH <7.10 may aggravate tissue hypoxia and lactic acidosis [5], [6]. However, sodium bicarbonate can induce acute hypercapnia, which increases intracellular acidosis and results in decreased myocardial contractility [7]. Sodium bicarbonate administration may also enhance pulmonary edema, especially in oliguric patients with volume overload. In addition, two short-term prospective studies did not demonstrate a hemodynamic benefit for sodium bicarbonate in patients with lactic acidosis [8], [9]. Still, alkaline therapy for acidosis correction is a matter of debate [10], [11], and no clinical studies have examined the effect of sodium bicarbonate administration on mortality. This study aimed to evaluate the cause and to explore long term hospital mortality rate of lactic acidosis and to investigate the effect of factors, including sodium bicarbonate, on death.

## Materials and Methods

### Patient Inclusion and Data Collection

We conducted this single center analysis from May 2011 through April 2012 at Dong-A University Hospital, Busan, Korea. We screened 207 patients with a serum CO2 concentration <20 mEq/L and a plasma lactic acid concentration. We defined lactic acidosis as a lactic acid level >30 mg/dL (3.3 mmol/L) with a high anion gap metabolic acidosis. High normal lactic acid level is 19.8 mg/dL (2.2 mmol/L) in our hospital laboratory. Therefore, we selected lactic acid level >3.3 mmol/L to exclude patients with equivocally high lactic acid level. Definitely patients just with hyperlactatemia without high anion gap metabolic acidosis were excluded. Finally, 103 patients were included in the analysis after excluding 104 patients without lactic acidosis. We retrospectively analyzed the patients’ medical records, including the patients’ underlying disease, laboratory findings, sodium bicarbonate administration, catecholamine use, ventilator care, continuous renal replacement therapy, survival and survival time. We checked the patients’ sex, age and vital signs, including mean arterial pressure, heart rate, blood temperature and respiratory rate, at the time of the lactic acidosis diagnosis. We specifically analyzed hemoglobin, albumin, liver function tests, C-reactive protein (CRP), blood urea nitrogen, and creatinine. This study was approved by the Dong-A University Hospital Institutional Review Board. Informed consent was waived because of the study’s retrospective design the data were analyzed anonymously. All clinical investigations were conducted in accordance with the guidelines of the 2008 Declaration of Helsinki.

### Analysis for Disease Severity

We used the SOFA (Sequential Organ Failure Assessment) and APACHE (Acute Physiology And Chronic Health Evaluation**s**)-II scores to estimate illness severity [12], [13]. Therefore, we analyzed arterial blood (PaO_2_, PaCO_2_, pH, bicarbonate), hematocrit, and white blood cell and platelet counts. We also checked procalcitonin as a marker of bacterial infection to confirm the infection severity at the time of diagnosis.

### Lactic Acid Level and Sodium Bicarbonate Group

We were able to check lactic acid levels at our hospital starting in May 2011, so we could diagnose lactic acidosis after that time. The plasma lactic acid level was measured using Roche/Hitachi 912/MODULAR P analyzers (ACN 040, Roche, Indianapolis, USA). We measured a follow-up lactic acid level at 24 hours after checking the initial lactic acid level.

We divided the patients into 2 groups depending on whether they received sodium bicarbonate >20 mEq during the investigational period. In addition, we selected 55 patients after excluding patients with an initial bicarbonate >20 mEq/L and SOFA score <8.0 to correct disease severity. And then we analyzed these 55 patients according to sodium bicarbonate administration.

### Statistical Analysis

The data are presented as the mean ± S.D. Shapiro-Wilk goodness-of-fit model was used to assure the data normality. Survival duration data was expressed as a median value with minimum and maximum values because of their non-normal distribution. Comparisons of non-parametric data according to survival and sodium bicarbonate administration were analyzed using a Mann-Whitney U test. Fisher’s exact test was used to compare categorical data between the 2 groups. Univariate and multivariate logistic regression analysis was used to identify factors that affected mortality. The variables included age, sex, lactic acid level, follow-up lactic acid level, albumin, APACHE II score, SOFA score, ventilator care and sodium bicarbonate administration. We tested collinearity using linear regression analysis and variance inflation factor for variables was less than 10. Also we separately analyzed factors associated with mortality using three different models for logistic regression analysis because SOFA score, APACHE II score and sodium bicarbonate administration are classically collinear and potentially collinear with each other. The variables included age, sex, lactic acid level, follow-up lactic acid level, albumin, ventilator care and APACHE II score in the model 1, SOFA score in the model 2 (excluding APACHE II score), and sodium bicarbonate administration in the model 3 (excluding APACHE II score and SOFA score). The effect of sodium bicarbonate on death was analyzed by Cox regression analysis. P values <0.05 were considered to be significant. All of the statistical calculations were performed using SPSS software version 19.0 (Statistical Package for Social Science version 19.0, SPSS, Inc, an IBM Company, Chicago, Illinois, USA).

## Results

### Patient Characteristics

**Figure 1:**
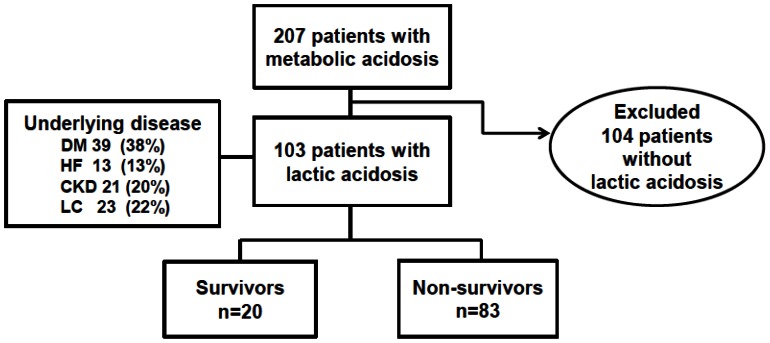
Overview of all of the patients screened and those included in the final analysis. DM, diabetes mellitus; HF, heart failure; CKD, chronic kidney disease, LC, liver cirrhosis.

Among the 207 patients with metabolic acidosis, we finally diagnosed 103 patients (49.9%) with lactic acidosis at our institution during the 1-year investigational period from 2011 to 2012. The male-to-female ratio was 72∶31, and the mean patient age was 66.1±11.4 years. The causes for lactic acidosis were sepsis (61.1%), hepatic failure (13.5%), cardiogenic shock (9.7%), malignancy (7.7%), seizure (4.8%) and other etiologies (2.8%). One patient had severe anemia as the cause of lactic acidosis. Of the enrolled patients, 37.9% had diabetes mellitus, 12.6% had heart failure, 20.4% had chronic kidney disease and 20.4% had liver cirrhosis (Figure 1). The percentage of patients with at least two comorbidities was 20.4%, and the percentage of patient with three comorbidities was 5.8%. The initial arterial pH was 7.30±0.16, PaCO2 was 29.1±12.0 mmHg, arterial bicarbonate was 14.2±5.0 mEq/L, lactic acid level was 77.3±42.7 mg/dL and anion gap was 19.7±6.9. The average SOFA score was 8.7±3.2, and the average APACHE II score was 23.0±6.2 (Table 1). Sodium bicarbonate was used in 69 patients (67.0%), and catecholamines were used in 66 patients (64.1%). Fifty patients (48.5%) received mechanical ventilation, 5 patients (4.9%) were treated with hemodialysis and 22 patients (21.4%) were treated with continuous renal replacement therapy (CRRT). Hemodialysis was treated with using dialysate solution composed of sodium chloride 21.89 g, potassium chloride 0.522 g, calcium chloride 0.643 g, magnesium chloride 0.356 g, sodium acetate 3.815 g and glucose 5.25 g among 100 mL. CRRT was treated with using dialysate solution composed of magnesium chloride 515 mg, sodium chloride 30.7 g, sodium bicarbonate 13.45 g, sodium lactate 1.68 g, calcium chloride 1.285 g among 5 L. The dosage of replacement and dialysate solutions was 1–2 L per hour in CRRT. Citrate was not used for anticoagulation in the renal replacement therapy.

**Table 1:** Patient characteristics according to survival.

	Total	Survivors	Non-survivors	p value
	(n = 103)	(n = 20)	(n = 83)	
Sex, male, n (%)	72 (70)	10 (50)	62 (74)	0.032
Age (years)	66.1±11.4	71.0±9.6	65.0±11.5	0.034
Cause of acidosis,n (%)				
sepsis	63 (61.1)	12 (60.0)	51 (61.4)	
hepatic failure	14 (13.5)	3 (15.0)	11 (13.3)	
cardiogenic shock	10 (9.7)	1 (5.0)	9 (10.8)	
malignancy	8 (7.7)	3 (15.0)	5 (6.0)	
seizure	5 (4.8)	1 (5.0)	4 (4.8)	
Others	3 (2.8)	0 (0)	3 (3.6)	
Initial pH	7.30±0.16	7.35±0.13	7.29±0.17	0.150
Initial bicarbonate (mEq/L)	14.2±5.0	14.7±3.5	14.0±5.3	0.598
Initial lactate(mg/dL)	77.3±42.7	55.4±27.5	82.6±44.2	0.010
Follow-up lactate(mg/dL)	70.2±49.9	40.3±18.9	80.1±53.1	0.008
Anion gap	19.7±6.9	17.6±5.2	20.2±7.2	0.125
Albumin (g/dL)	3.1±0.6	3.4±0.5	3.1±0.6	0.028
BUN (mg/dL)	41.6±24.4	35.9±22.6	42.9±24.8	0.249
Creatinine (mg/dL)	2.5±2.1	2.4±1.9	2.5±2.1	0.811
CRP (mg/dL)	11.9±9.8	9.3±9.0	12.5±9.9	0.187
Procalcitonin (ng/mL)	31.9±56.5	24.9±53.6	33.7±57.5	0.569
SOFA	8.7±3.2	6.2±2.8	9.4±3.0	0.000
APACHE II	23.0±6.2	20.3±5.8	23.6±6.1	0.031
Bicarbonate use,n (%)	69 (67.0)	8 (40.0)	61 (73.5)	0.006
Ventilator use, n (%)	50 (48.5)	5 (25.0)	45 (54.2)	0.017
Catecholamine use,n (%)	66 (64.1)	6 (45.0)	60 (72.3)	0.001

n, number, CRP, C-reactive protein; SOFA, sepsis related organ failure assessment; APACHE II, acute physiologic and chronic health evaluation.

Two patients were at emergency room and all patients were dead. Forty eight patients (mortality rate was 79.2%) were at ward and 53 patients (mortality rate was 81.1%) were at intensive care unit (ICU).

### Patient Characteristics According to Survival

**Figure 2:**
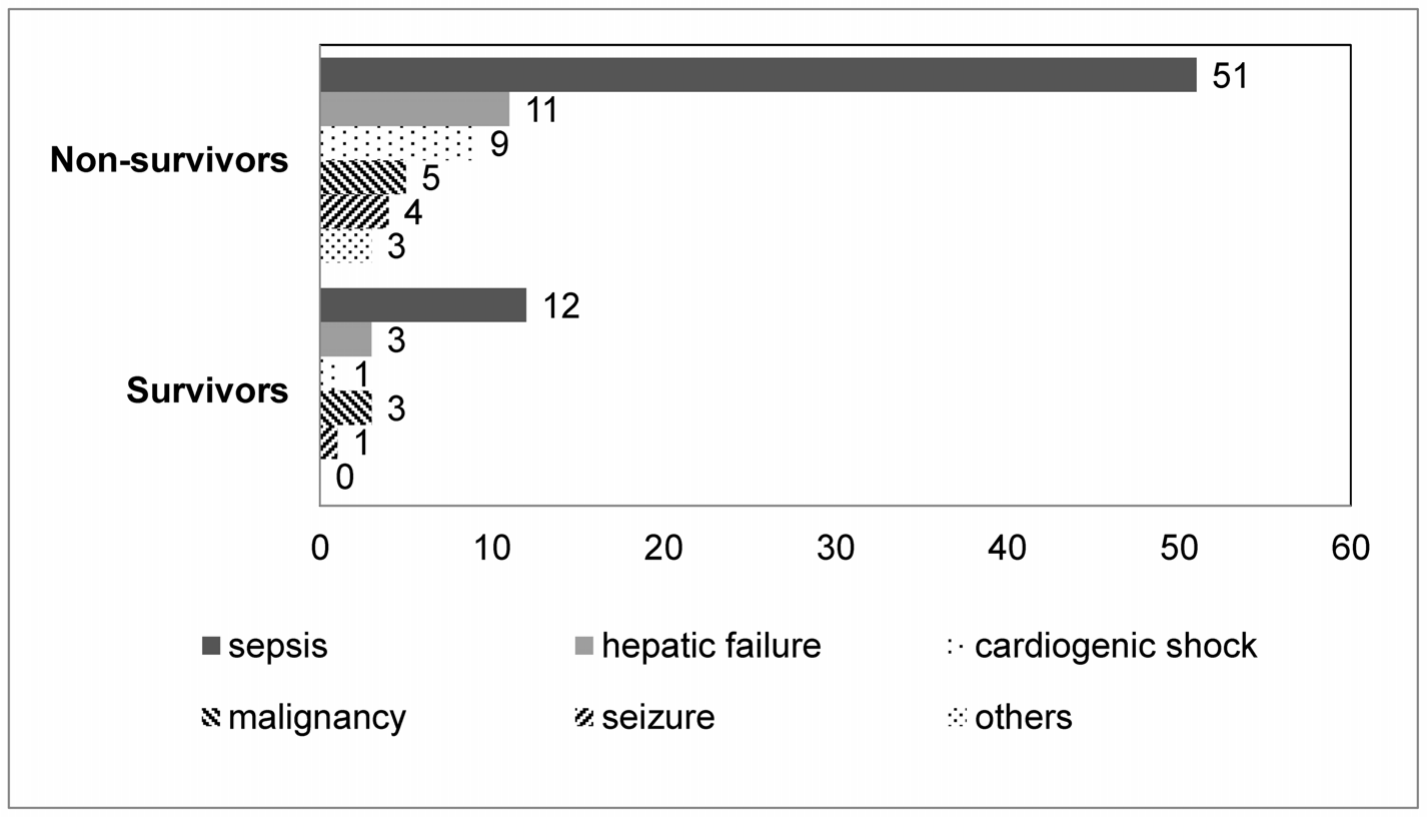
Causes of lactic acidosis and mortality.

Eighty-three patients (80.6%) died from sepsis (61.4%), hepatic failure (13.2%), cardiogenic shock (10.8%) and other causes (Figure 2). The median survival time was 2 days (1–43 days) for the non-survivors. The non-survivors were more likely to have received sodium bicarbonate (p = 0.006), catecholamines (p = 0.001), and mechanical ventilation (p = 0.017), and a higher percentage was male (p = 0.032) and younger (p = 0.034) compared to the survivors (Table 2). The non-survivors had lower initial albumin levels (p = 0.028), higher initial lactic acid levels (p = 0.010), higher lactic acid levels at follow-up (p = 0.008), higher SOFA scores (p<0.001) and higher APACHE II scores (p = 0.031) compared to the survivors (Table 2). There were no significant differences in the initial pH, initial bicarbonate, serum creatinine, CRP, procalcitonin and the causes of lactic acidosis between the survivors and non-survivors.

**Table 2:** Patient characteristics according to sodium bicarbonate administration (n = 103).

Variable	Bicarbonate use	Non-use	p value
	(n = 69)	(n = 34)	
Sex, male, n (%)	48 (69.6)	24 (70.6)	1.000
Age, years	65.8±11.3	66.7±11.7	0.705
Initial pH	7.267±0.160	7.371±0.140	0.002
Initial bicarbonate (mEq/L)	13.1±4.6	16.4±5.2	0.001
Initial lactate (mg/dL)	84.3±45.5	63.0±32.5	0.016
Follow-up lactate (mg/dL)	78.7±54.2	46.8±23.7	0.033
Anion gap	20.7±7.4	17.9±5.5	0.053
Albumin (g/dL)	3.1±0.6	3.2±0.5	0.063
BUN (mg/dL)	43.6±24.6	37.5±23.8	0.236
Creatinine (mg/dL)	2.5±1.9	2.4±2.4	0.809
CRP (mg/dL)	12.1±10.8	11.4±7.6	0.698
Procalcitonin (ng/mL)	36.9±61.1	21.0±44.2	0.228
SOFA	9.5±3.1	7.0±2.9	0.000
APACHE II	24.0±6.5	20.8±4.9	0.012
Ventilator use, n (%)	43 (62.3)	7 (20.6)	0.000
Non-survivor, n (%)	61 (88.4)	22 (64.7)	0.007

n, number; CRP, C-reactive protein; SOFA, sepsis related organ failure assessment; APACHE II, acute physiologic and chronic health evaluation.

### Patient Characteristics According to Sodium Bicarbonate Administration

Patients who received sodium bicarbonate had lower arterial pH (p = 0.002), lower bicarbonate levels (p = 0.001), higher lactic acid levels (p = 0.016), higher follow-up lactic acid levels (p = 0.033), higher SOFA scores (p<0.001) and higher APACHE II scores (p = 0.012) than the patients who did not receive sodium bicarbonate. The sodium bicarbonate group was more likely to have received mechanical ventilation (p<0.001) and had a higher mortality (p = 0.007) (Table 2). There were no significant differences in age, male gender, albumin, creatinine, CRP and procalcitonin according to sodium bicarbonate administration.

In the subgroup analysis after excluding 48 patients who had SOFA scores <8 and initial bicarbonate levels >20 mEq/L, the percentage of patients with ventilator care (p = 0.020) and percentage of non-survivors (p = 0.049) was greater in the patients treated with sodium bicarbonate despite having similar laboratory data compared to the patients who did not receive sodium bicarbonate (Table 3).

**Table 3:** Clinical characteristics according to sodium bicarbonate administration after excluding patients with an initial bicarbonate >20 mEq/L and SOFA score <8.0 (n = 55).

Variable	Bicarbonate use	Non-use	p value
	(n = 46)	(n = 9)	
Sex, male, no. (%)	33 (71.7)	6 (66.7)	0.710
Age, years	65.3±12.0	64.6±12.0	0.870
Initial pH	7.244±0.168	7.296±0.172	0.406
Initial bicarbonate (mEq/L)	12.3±3.7	13.1±3.1	0.548
Initial lactate (mg/dL)	91.6±42.1	77.9±35.9	0.366
Follow-up lactate (mg/dL)	86.5±50.7	51.2±28.3	0.142
Anion gap	22.4±7.0	19.2±5.6	0.192
Albumin (g/dL)	3.1±0.6	3.1±0.6	0.965
BUN (mg/dL)	47.5±25.2	51.6±19.3	0.787
Creatinine (mg/dL)	2.9±2.2	3.9±3.0	0.254
CRP (mg/dL)	12.3±11.5	10.1±8.7	0.594
Procalcitonin (ng/mL)	34.5±55.3	47.8±74.6	0.579
SOFA	11.0±2.2	10.1±1.5	0.236
APACHE II	23.9±3.6	25.7±6.6	0.444
Ventilator use, n (%)	32 (69.6)	2 (22.2)	0.020
Non-survivor, n (%)	43 (93.5)	6 (66.7)	0.049

n, number; CRP, C-reactive protein; SOFA, sepsis related organ failure assessment; APACHE II, acute physiologic and chronic health evaluation.

### Factors Associated with Mortality

**Figure 3:**
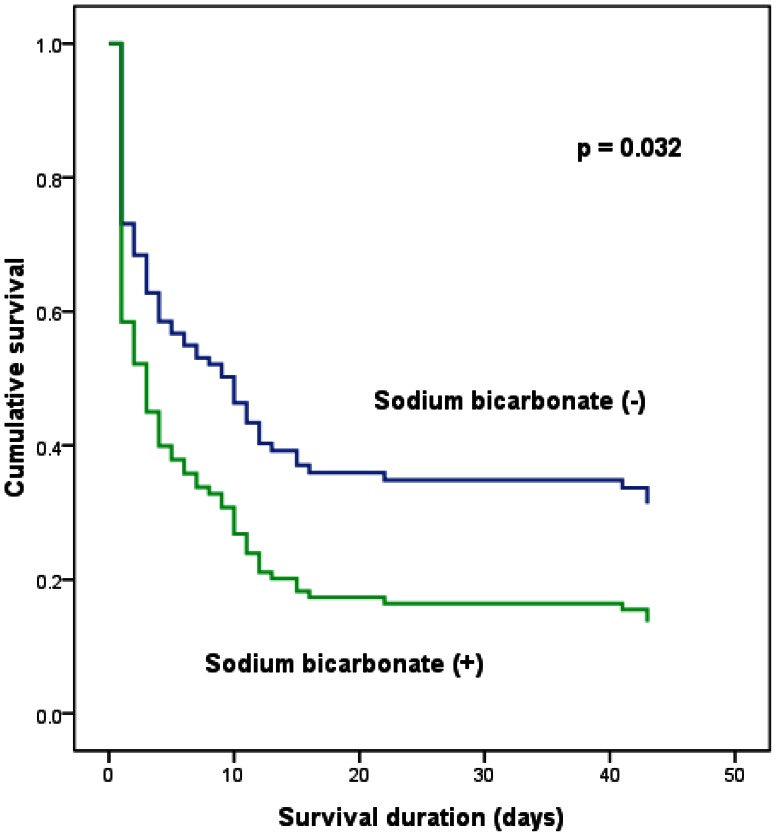
Cumulative survival curves for mortality according to sodium bicarbonate administration.

Age, male gender, albumin level, initial lactic acid level, follow-up lactic acid level, APACHE II score, SOFA score, mechanical ventilation and sodium bicarbonate administration were associated with mortality in the univariate logistic regression analysis. The SOFA score (Exp (B) = 3.02, 95% confidence interval (C.I.) = 1.23–7.42, p = 0.016) and sodium bicarbonate administration (Exp (B) = 5.83, 95% C.I. = 1.00–251.47, p = 0.050) were independent factors for mortality in the multiple logistic regression analysis (Table 4). There was no independent factor for mortality in the model 1. SOFA score (Exp (B) = 1.72, 95% confidence interval (C.I.) = 1.12–2.63, p = 0.013) was independent factor for mortality in the model 2 and sodium bicarbonate administration (Exp (B) = 6.27, 95% C.I. = 1.10–35.78, p = 0.039) was independent factor for mortality in the model 3. Sodium bicarbonate administration ((Exp (B) = 1.71, 95% C.I. = 1.05–2.80, p = 0.032) was associated with mortality in the Cox regression analysis model (Figure 3).

**Table 4:** Logistic regression analysis for factors that affect mortality.

	Univariate		Multivariate	
Variable	Exp(B) (95% CI)	p value	Exp(B) (95% CI)	p value
Sex, male	2.95 (1.08–8.08)	0.035	1.55 (1.00–24.68)	0.756
Age	0.95 (0.90–1.00)	0.040	0.71 (0.47–1.08)	0.110
Albumin	0.35 (0.13–0.92)	0.032	112.6 (0.1–109347.3)	0.178
SOFA	1.50 (1.21–1.86)	0.000	3.02 (1.23–7.42)	0.016
APACHE II	1.10 (1.01–1.21)	0.035	0.69 (0.46–1.04)	0.078
Initial lactate	1.03 (1.00–1.05)	0.016	1.01 (0.97–1.05)	0.705
Follow-up lactate	1.03 (1.00–1.05)	0.020	1.02 (1.00–1.06)	0.222
Bicarbonate use	4.16 (1.50–11.52)	0.006	15.83 (1.00–251.47)	0.050
Ventilator use	3.55 (1.18–10.68)	0.024	0.91 (0.10–8.56)	0.934

SOFA, sepsis related organ failure assessment; APACHE II, acute physiologic and chronic health evaluation; Exp(B), exponentiation of the B coefficient, which is an odds ratio; CI, confidence interval.

## Discussion

In this retrospective study, we observed that sodium bicarbonate administration in patients with lactic acidosis was associated with increased mortality. Clearly, patients treated with sodium bicarbonate had more severe disease, lower initial bicarbonate levels and higher initial lactic acid levels compared to the patients who did not receive sodium bicarbonate. However, in the subgroup analysis after excluding patient with lower disease severity and the higher initial bicarbonate level, the patients treated with sodium bicarbonate still had a higher mortality rate compared to the patients who did not receive sodium bicarbonate. Definitely, severe metabolic acidosis caused by increased lactate production or decreased lactate clearance may produce hemodynamic instability. But our results imply that sodium bicarbonate administration just for correcting metabolic acidosis without decreasing lactate production or increasing lactate clearance may negatively affect survival in patients with metabolic acidosis caused by lactic acidosis. To our knowledge, this study is the first report to show that sodium bicarbonate administration may harmfully affect outcomes in patients with lactic acidosis.

Acidosis may inhibit lactic acid production by reducing the enzyme activity of phosphofructokinase [11], [14]. Therefore, a clumsy correction of metabolic acidosis using sodium bicarbonate may increase lactic acid production by inhibiting the compensatory response. Practically many physicians easily prescribe sodium bicarbonate to prevent progression of acidemia in ICU because they worry about the status of acidemia. In addition, nephrologists try to correct metabolic acidosis even though normal pH with metabolic acidosis, because sodium bicarbonate therapy is associated with an improvement in renal function and prevents the progression of chronic kidney disease [15]. A recent report showed that 67% of critical care physicians start alkaline solutions for patients with lactic acidosis when the pH is 7.2 [16]. Sodium bicarbonate was infused even when patients did not have pH lower than 7.15 in our patients with lactic acidosis. Notably, the average initial pH was 7.27, and the percentage of patients with an initial pH >7.15 was greater than 75% in the patients treated with sodium bicarbonate. Our results showed that the follow-up mean lactic acid level was less decreased by at least 10 mg/dL in the patients who received sodium bicarbonate compared to those who did not. Therefore, correcting metabolic acidosis with sodium bicarbonate may negatively affect recovery from lactic acidosis, especially in patients without severe acidemia. It should be considered to warn that sodium bicarbonate administration is not helpful, and is even harmful, in patients with lactic acidosis of their pH >7.2 in clinical practice. Further prospective and retrospective studies are necessary to clarify the effect of sodium bicarbonate administration at several point of pH in patients with lactic acidosis.

Another harmful effect of sodium bicarbonate in lactic acidosis can be explained by worsening acidosis due to an increasing PaCO_2_. Infused sodium bicarbonate combines with hydrogen ions to form H_2_CO_3_. H_2_CO_3_ dehydrates to H_2_O and CO_2_. Therefore, sodium bicarbonate administration physiologically increases CO_2_. If adequate ventilation is prohibited, sodium bicarbonate administration worsens the acidemia caused by the combination of respiratory acidosis and lactic acidosis. Furthermore, even with adequate ventilation, increased CO_2_ due to sodium bicarbonate worsens intracellular acidosis in severely ill patients with circulatory failure, although sodium bicarbonate increases the arterial pH in the short term [7], [17]. In this study, sodium bicarbonate was not administered for two patients with PaCO_2_>50 mmHg, although they had an initial pH <7.15. However, one of two patients with sepsis maintained pH >7.15 just with mechanical ventilation by maintaining PaCO_2_<20 mmHg and finally recovered from lactic acidosis without sodium bicarbonate administration. One patient recovered from heart failure and lactic acidosis, but developed alkalemia in the absence of sodium bicarbonate administration. Alkalemia was induced by the respiratory alkalosis to mechanical ventilation with a high minute volume and increased bicarbonate conversion from lactic acid. Further investigations are necessary to evaluate whether lower PaCO_2_ using mechanical ventilation can overcome acidemia in patients with lactic acidosis.

The causes of lactic acidosis can be classified as type A, which is associated with marked tissue hypoperfusion, such as shock, or type B, which is not associated with tissue hypoxia. The most common cause of severe lactic acidosis is sepsis [2]. Sepsis was the most common cause of lactic acidosis, and the most common cause of mortality in this study. We only had two days to improve lactic acidosis because the median survival time was just two days in this and other studies [2]. Therefore, intensive management is necessary to increase the survival rate in patients with sepsis and lactic acidosis.

Jansen and Bakker et al. showed that the SOFA score is closely associated with the lactic acid level in patients in the ICU [18]. The SOFA score is composed of six components. Two components are related to tissue hypoperfusion, and another two are associated with kidney and liver function. Therefore, the SOFA score accurately reflects lactate production due to hypoxia and lactate clearance. The SOFA score, which can be easily calculated, was independent factor for mortality, and the APACHE II score, which has fourteen components, was correlated with mortality in this study. Further studies are necessary to confirm the role of the SOFA score in predicting mortality in patients with lactic acidosis.

This study has some limitations because the sample size is small, and the study design is retrospective. In addition, non-calibration and potential inaccuracy of APACHE II score to predict death and a false severity correction make a limited interpretation. Despite these limitations, we found that sodium bicarbonate administration may be harmful in patients with lactic acidosis. Further prospective studies are necessary to confirm the effect of sodium bicarbonate on mortality in patients with lactic acidosis.

In summary, we have found that lactic acidosis is a common cause of high anion gap metabolic acidosis and has a very high mortality rate especially in patients with sepsis. Therefore, the lactic acid level should be checked to evaluate the cause of metabolic acidosis when patients present with high anion gap metabolic acidosis especially those with sepsis. The SOFA score is an independent factor for predicting mortality and the initial and follow-up lactic acid levels are associated with mortality in patients with lactic acidosis. In addition, sodium bicarbonate should be prescribed with caution in cases of lactic acidosis because sodium bicarbonate administration may affect mortality.
